# WT1‐Targeted Oral Bifidobacterium longum Vaccine Enhances Checkpoint Blockade Efficacy in Pancreatic Cancer

**DOI:** 10.1002/advs.202524323

**Published:** 2026-07-07

**Authors:** Taiki Yamazaki, Aori Minami, Misako Hirota, Mirei Hirano, Ryota Sato, Atsuki Honma, Hideto Ueki, Toshiro Shirakawa

**Affiliations:** ^1^ Laboratory of Translational Research for Biologics, Department of Advanced Medical and Pharmaceutical Sciences Kobe University Graduate School of Science, Technology and Innovation Kobe Japan; ^2^ Department of Urology Kobe University Graduate School of Medicine Kobe Japan

**Keywords:** cancer, cancer therapy, immunotherapy, pancreatic cancer

## Abstract

Pancreatic ductal adenocarcinoma (PDAC) remains largely unresponsive to immune checkpoint inhibitors (ICI) due to its profoundly immunosuppressive tumor microenvironment. To overcome this resistance, we evaluated an oral WT1‐targeted cancer vaccine using a recombinant Bifidobacterium longum strain (*B. longum* 420) engineered to display a partial WT1 antigen. Oral immunization with *B. longum* 420 activated WT1‐associated cellular immunity, increasing IL‐12–producing CD45^+^ cells and Th1/cytotoxic cytokine‐producing T cells, and enhancing splenocyte cytotoxic activity against Pan02 cells. In a syngeneic Pan02 tumor model, the combination of *B. longum* 420 with anti‐PD‐1 and anti‐CTLA‐4 antibodies resulted in stronger antitumor effects and improved survival compared with each monotherapy, without marked body weight loss. An empty‐vector control strain did not further enhance the efficacy of dual checkpoint blockade. Tumor analyses showed increased intratumoral CD8^+^ T cell density and expansion of effector, central memory, and PD‐1^+^ Ki‐67^+^ proliferating CD8^+^ subsets, together with treatment‐dependent differences in intratumoral CD4^+^ T cell subset composition. These findings demonstrate that an oral WT1‐targeted Bifidobacterium vaccine can potentiate dual checkpoint blockade and promote a more immunologically active T cell landscape in PDAC. To our knowledge, this is the first demonstration of this combination strategy in a syngeneic pancreatic cancer model.

## Graphical abstract

Oral WT1‐targeted Bifidobacterium longum vaccine promotes WT1‐associated cellular immunity and enhances anti‐PD‐1/anti‐CTLA‐4 efficacy in pancreatic cancer.

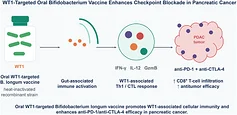

## 1 Introduction

Pancreatic ductal adenocarcinoma (PDAC) remains one of the most lethal malignancies, with a 5‐year survival rate of less than 13%. Despite advances in surgical techniques and chemotherapy, the prognosis for PDAC patients remains poor, highlighting an urgent need for novel therapeutic approaches [1]. While ICIs, particularly anti‐PD‐1 and anti‐CTLA‐4 antibodies, have revolutionized cancer treatment in various malignancies, PDAC has shown limited response to ICI monotherapy, with response rates typically below 5% [2, 3, 4]. This resistance is attributed to the immunosuppressive tumor microenvironment of PDAC, characterized by dense desmoplastic stroma, poor T cell infiltration, and dominance of immunosuppressive cells, creating a “cold” tumor phenotype [5, 6, 7, 8]. Therefore, strategies to enhance tumor immunogenicity and promote T cell infiltration are crucial for improving the efficacy of ICIs in PDAC.

Cancer vaccines represent a promising approach to stimulate tumor‐specific T cell infiltration.　In particular, cancer vaccines utilizing WT1 are showing great promise due to its overexpression in various solid malignancies, including PDAC, and its high immunogenicity [9, 10, 11, 12]. Recent reports have shown that several WT1‐targeted therapeutic approaches, including peptide vaccines and dendritic cell vaccines, exhibit enhanced efficacy when combined with chemotherapy or other immunotherapies. However, a WT1 cancer vaccine that achieves sufficient therapeutic efficacy has not yet been established [13, 14, 15, 16].

Previously, we constructed a recombinant Bifidobacterium longum displaying a partial mouse WT1 protein (*B. longum* 420). We previously demonstrated that *B. longum* 420 could induce multiple WT1 epitope‐specific cellular immunity and significantly inhibit tumor growth in WT1‐expressing mouse prostate cancer, renal cell carcinoma, and bladder cancer models [17, 18, 19]. On the basis of the reports that approximately 70% of pancreatic cancer expresses WT1 protein [10, 20], we explored whether this WT1‐targeted oral vaccine platform can overcome the immunotherapy‐refractory nature of PDAC, particularly in combination with dual checkpoint blockade.

Notably, PDAC represents a particularly stringent and immunotherapy‐refractory context compared with the tumor models evaluated in our previous studies. Its profoundly immunosuppressive tumor microenvironment, limited T‐cell infiltration, and desmoplastic stroma contribute to poor responsiveness to ICI therapy. Therefore, it remains unclear whether the WT1‐targeted oral Bifidobacterium vaccine platform can sensitize PDAC to dual checkpoint blockade and promote a more immunologically active tumor microenvironment.

This study aimed to evaluate whether combining an oral cancer vaccine with ICIs, which show limited efficacy against PDAC, could enhance therapeutic effects. We evaluated cytokine production, tumor‐infiltrating T cells, and the synergistic effects of *B. longum* 420 combined with anti‐PD‐1 and anti‐CTLA‐4 antibodies.

## 2 Materials and Methods

### 2.1 Recombinant Bifidobacterium

A recombinant *B. longum* 420 strain that expresses a partial murine‐WT1 protein (a.a. 117–419) fused to galacto‐N‐biose/lacto‐N‐biose I binding protein (GL‐BP) was constructed by shuttle‐vector electroporation into *B. longum* 105‐A (the Japan Collection of Microorganisms, RIKEN Bioresource Center, Tsukuba, Japan) in our previous study [21]. An empty‐vector control strain, *B. longum* 2012, was generated in parallel and used as an antigen‐negative control. *B. longum* 2012 was cultured and heat‐inactivated in the same manner as *B. longum* 420. GL‐BP is a membrane protein in the ATP‐binding cassette transporter on the wild‐type *B. longum* cell wall, which we used as an anchor to display antigen on the bacterial cell surface. *B. longum* 420 was anaerobically cultured in Gifu anaerobic medium (Nissui, Tokyo, Japan) with 50 µg/mL spectinomycin at 37°C. After cultivation, bacteria were heated for inactivation at 65°C for 5 min. Complete inactivation was verified by anaerobic culture on agar plates, confirming no colony formation (Figure S1). Because *administered bacteria were* heat‐inactivated prior to oral administration, they were not expected to replicate or colonize in the gastrointestinal tract. For each animal experiment, heat‐inactivated *B. longum* preparations were independently cultured and prepared on different days. The dosing amount was standardized to 1 × 10^9^ CFU‐equivalent for each batch, as determined before heat inactivation.

### 2.2 Cell Line

Pan02, a mouse PDAC cell line expressing murine‐WT1 protein derived from C57BL/6, was purchased from Division of Cancer Treatment and Diagnosis (DCTD) Tumor Repository, National Cancer Institute (NCI, Frederick, MD, USA; RRID: CVCL_D627) and maintained in Roswell Park Memorial Institute 1640 (RPMI‐1640) medium supplemented with 10% fetal bovine serum (Sigma‐Aldrich Japan, Tokyo, Japan) and 1% penicillin–streptomycin (Nacalai Tesque, Kyoto, Japan). Cells were confirmed to be contamination‐free prior to use. The overexpression of WT1 protein in Pan02 was confirmed by western blotting analysis (Figure S2).

### 2.3 Immunization with *B. longum* 420

Female C57BL/6J mice, 5 weeks of age, were orally administered 100 µL of PBS with or without 1.0 × 10^9^ CFU‐equivalent of heat‐inactivated *B. longum* 420, 5 times a week for a week (days 0–4), using a feeding needle. After vaccination, mice were euthanized, and spleen cells were isolated for evaluation of immune responses by the following in vitro assays.

### 2.4 Intracellular Cytokine Staining for Immunized Splenocytes

Isolated splenocytes were maintained in RPMI‐1640 medium supplemented with 10% FBS, 10 mmol/L HEPES, 100 U/mL penicillin, 100 µg/mL streptomycin, 1 mmol/L nonessential amino acids, 50 µmol/L 2‐mercaptoethanol, and 1 mmol/L sodium pyruvate. A total of 2.0 × 10^6^ splenocytes were cultured with 2.0 × 10^5^ mitomycin C‐treated Pan02 cells in vitro. For mitomycin C treatment, Pan02 cells were incubated with mitomycin C (200 µg/mL) for 1 h at 37°C with shaking and then washed three times with complete culture medium before use. After 6 h of culture, GolgiStop (BD Biosciences, San Jose, CA, USA) was added to the culture, and the cells were then cultured for an additional 12 h. For intracellular cytokine staining (ICCS), cells were collected and processed using a BD Cytofix/Cytoperm Plus Fixation/Permeabilization Kit (BD Biosciences) according to our previous study [21]. Briefly, collected cells were blocked and stained with PE‐Cy5.5‐anti‐mouse CD3 (Miltenyi Biotec, Bergisch Gladbach, Germany), Brilliant Violet 510‐anti‐mouse CD45, FITC‐anti‐mouse CD4, and Alexa Fluor 700‐anti‐mouse CD8a, Brilliant Violet 421‐anti‐mouse CD69 antibodies (BioLegend, San Diego, CA, USA). Then, the washed cells were fixed and permeabilized with BD Fixation/Permeabilization solution. Cells were washed and stained with Brilliant Violet 605‐anti‐mouse IFN‐γ, PE‐Cy7‐anti‐mouse TNF‐α, APC‐anti‐mouse IL‐12, PE‐Dazzle‐594‐anti mouse Granzyme B or PE‐anti‐mouse IL‐2 (BioLegend) for intracellular staining. Stained cells were analyzed using a MACSQuant Analyzer 16 (Miltenyi Biotec).

### 2.5 WT1 Tetramer Staining for Immunized Splenocytes

Isolated splenocytes were maintained in RPMI‐1640 medium supplemented with 10% FBS, 10 mmol/L HEPES, 100 U/mL penicillin, 100 µg/mL streptomycin, 1 mmol/L nonessential amino acids, 50 µmol/L 2‐mercaptoethanol, and 1 mmol/L sodium pyruvate. A total of 2.0 × 10^6^ splenocytes were cocultured with 2.0 × 10^5^ mitomycin C‐treated Pan02 cells in vitro for 7 days. After coculture, cells were collected, washed, and blocked with an Fc receptor blocking reagent. Cells were stained with PE‐Cy5.5‐anti mouse CD3, Alexa Fluor 700‐anti mouse CD8a (BioLegend), and PE‐ T‐Select H‐2D b WT1 126 to 134 Tetramer (Medical and Biological Laboratories, Tokyo, Japan). Stained cells were analyzed using a MACSQuant Analyzer 16 (Miltenyi Biotec). WT1 tetramer‐positive cells were quantified as the percentage of WT1 tetramer‐positive cells among CD8^+^ T cells.

### 2.6 Measurement of Cytotoxic T‐Cell Activity

A total of 3.0 × 10^7^ splenocytes were cultured with 3.0 × 10^6^ mitomycin C‐treated Pan02 cells in the presence of 40 ng/mL IL‐2 in vitro for 4 days to generate effector cells. After culture, effector cells were cocultured with Pan02 at a ratio of 80:1, 40:1, and 10:1 for 8 h. Then, the culture supernatant was collected, and specific CTL activity was measured using an LDH Cytotoxicity Assay Kit (CytoTox 96 Non‐Radioactive Cytotoxicity Assay; Promega) according to the manufacturer's instructions. The percentage of specific killing was calculated by the following formula: percentage‐specific killing = (experimental release–effector spontaneous release–target spontaneous release)/(target maximum release–target spontaneous release) × 100.

### 2.7 Animal Experiments for Combination Therapy

To evaluate the in vivo antitumor activity of oral *B. longum* 420 in combination with immune checkpoint inhibitors (ICIs) against PDAC, we employed a syngeneic subcutaneous pancreatic tumor model using Pan02 cells. Under isoflurane anesthesia, 1.0 × 10^6^ Pan02 cells were subcutaneously injected into 5‐week‐old female C57BL/6J mice on day 0. Tumor growth and survival were evaluated in two independent experiments: one experiment for tumor volume measurement (total n = 42; group sizes n = 11 or 10) and a separate experiment for Kaplan–Meier survival analysis (total n = 28; n = 7 per group). On day 14, tumor‐bearing mice were randomly assigned to the following four treatment groups: (i) *B. longum* 420 + ICIs, (ii) *B. longum* 420, (iii) PBS + ICIs, and (iv) PBS. Oral administration of PBS or *B. longum* 420 was performed five times per week for three weeks (days 14–18, 21–25, and 28–32) using a feeding needle. Gastrointestinal residence of the administered bacteria was not directly quantified over time. For ICI treatment, anti‐mouse PD‐1 (clone RMP1‐14, Bio X Cell, Lebanon, NH, USA) and anti‐mouse CTLA‐4 (clone 9H10, Bio X Cell) antibodies were administered intraperitoneally on days 15, 18, 22, 25, 29, and 32 at a dose of 200 µg each antibody in 100 µL per injection. Tumor volume was measured by the calculation formula of (longest diameter) × (shortest diameter)2 × 0.5. Mice were euthanized when tumors exceeded 20 mm in diameter, and Kaplan–Meier survival curves were generated.

### 2.8 Immunohistochemical Study

Another set of mice was injected with 1.0 × 10^6^ Pan02 and treated by the same method described above. Tumors were resected on day 20 and fixed with 4% paraformaldehyde‐PBS and embedded in paraffin. Paraffin embedded tumor tissue sections were deparaffinized and rehydrated. Antigen retrieval was performed in Bond epitope retrieval buffer (pH6.0 for CD8, pH9.0 for CD4; Leica Microsystems, Wetzlar, Germany) at 98°C for 20 min. Immunohistochemical staining was performed in an automatic tissue processor (Leica Microsystems Bond) according to the manufacturer's standard protocol. Briefly, tissue sections were incubated at RT for 15 min with rabbit anti‐mouse CD4 antibody (1:1,000, Abcam, Cambridge, UK) or rabbit anti‐mouse CD8a antibody (1:2000, Abcam). After washing, sections were incubated with horseradish peroxidase‐conjugated secondary antibodies. After washing, sections were incubated with 3,3′‐diaminobenzidine and were counterstained with hematoxylin. Resulting tissue slides were observed using a BZ‐X710 microscope (Keyence, Osaka, Japan). Cell densities were quantified using QuPath. The ROI was defined as the entire tissue area within each image (excluding blank background), and positive cells were reported as cells/mm^2^. For each mouse, three randomly selected fields of view were analyzed and averaged.

### 2.9 Tumor‐Infiltrating T Cell Analysis

Under the same experimental conditions as described above, mice were euthanized after one week of treatment, and tumors were collected for analysis on day 22 (n = 7 or 6). Tumor tissues were dissociated using a Tumor Dissociation Kit, mouse (Miltenyi Biotec) to obtain single‐cell suspensions. The collected cells were blocked and stained with PE‐Cy5.5–anti‐mouse CD3 (Miltenyi Biotec), Brilliant Violet 510–anti‐mouse CD45, FITC–anti‐mouse CD4, Alexa Fluor 700–anti‐mouse CD8a, PE‐Cy7–anti‐mouse PD‐1, Brilliant Violet 570–anti‐mouse CD62L, Brilliant Violet 605–anti‐mouse CD44 antibodies (BioLegend), and Brilliant Violet 421–anti‐mouse CD25 (BioLegend). After washing, cells were fixed and permeabilized using the BD Fixation/Permeabilization Solution and subsequently stained intracellularly with PE–anti‐mouse Ki‐67 (BioLegend), APC–anti‐mouse FoxP3 (Miltenyi Biotec), and PE–anti‐mouse T‐bet (BioLegend). Stained cells were analyzed using a MACSQuant Analyzer 16 (Miltenyi Biotec).

### 2.10 Statistical Analysis

No outliers were excluded from the analyses. For ICCS and TIL experiments, data were converted to proportions and expressed as percentages of the indicated parent population. Data are presented as mean ± SEM, and n denotes the number of mice, as specified in each figure legend. All statistical tests were performed as two‐sided, and differences were considered statistically significant when *p <* 0.05. For comparisons among multiple groups, one‐way ANOVA followed by the Tukey–Kramer method was employed. For comparisons between two groups, a two‐sided Student's t‐test was used. Survival curves were generated using the Kaplan–Meier method and compared between groups using the log‐rank (Mantel–Cox) test. All statistical analyses were performed with EZR (version 1.70) (Saitama Medical Center, Jichi Medical University, Saitama, Japan), which is a graphical user interface for R (The R Foundation for Statistical Computing, Vienna, Austria) and a modified version of R Commander designed to add statistical functions frequently used in biostatistics.

## 3 Results

### 3.1 *B. longum 420* Induced WT1‐Related Immune Responses Measured by Intracellular Cytokine Staining and WT1 Tetramer Staining

To evaluate T cell activation by *B. longum* 420, ICCS was performed using splenocytes restimulated with mitomycin C‐treated Pan02 cells. In the *B. longum* 420 group, IL‐12‐producing cells, IFN‐γ and TNF‐α‐producing cells among CD4^+^ T cells, and Granzyme B, IFN‐γ, and TNF‐α‐producing cells among CD8^+^ T cells were significantly higher than those in the PBS group (*p <* 0.05, *p <* 0.01). Additionally, CD69^+^ cells among CD4^+^ T cells, IL‐2‐producing cells, and CD69^+^ cells among CD8^+^ T cells also tended to increase compared with the PBS group (Figure 1) (Figure S3). WT1 tetramer staining of splenocytes did not show a clear difference in the frequency of WT1 tetramer‐positive CD8^+^ T cells between the PBS and *B. longum* 420 groups (Figure S4).

**FIGURE 1:**
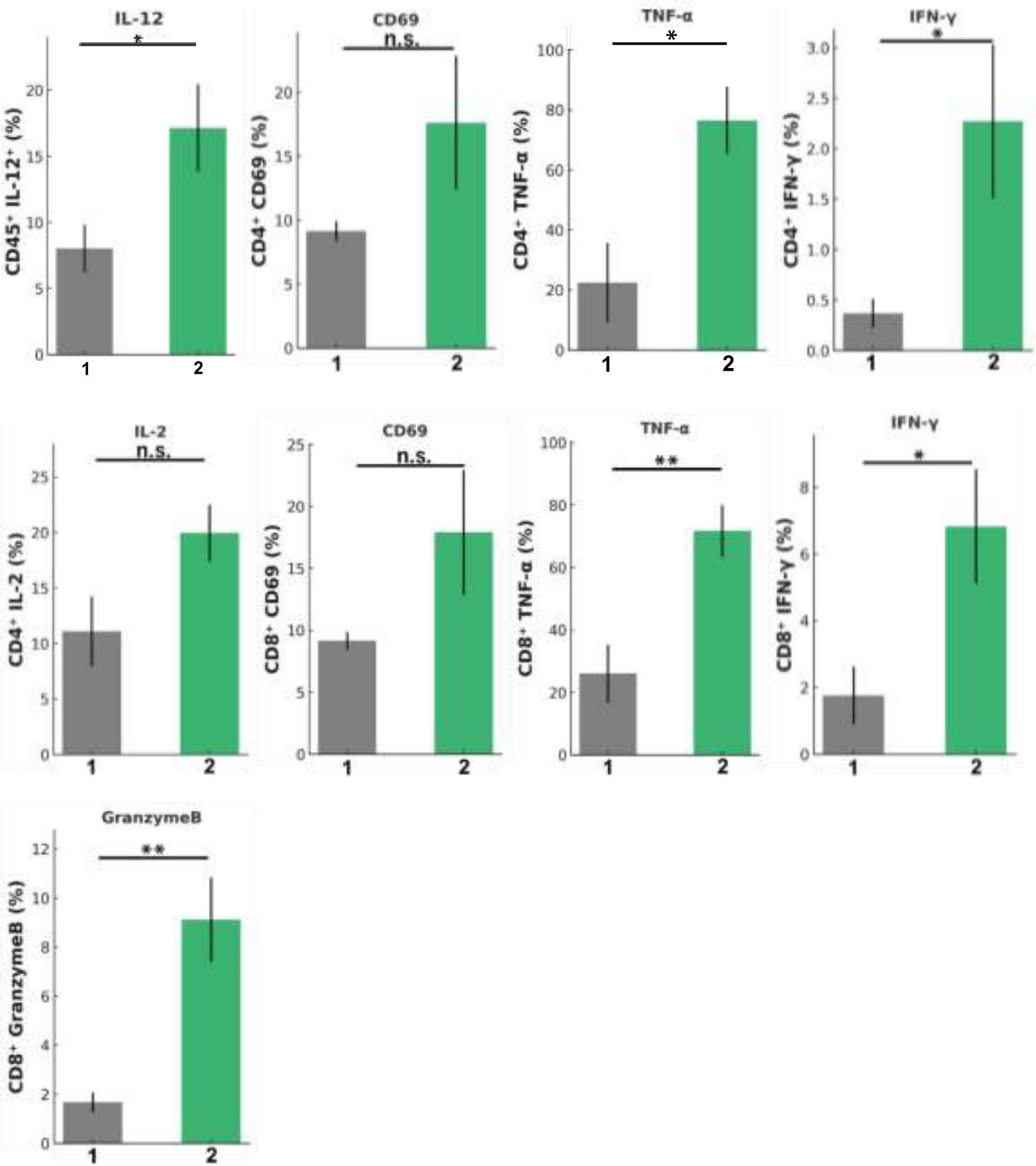
Frequency of Activated Splenocytes and Their Cytokine Production Induced by Oral Vaccination of *B. longum* 420. Splenocytes were harvested from vaccinated mice and restimulated ex vivo with mitomycin C–treated Pan02 cells, followed by flow‐cytometric analysis of activation and cytokine production (gating strategy is shown in Figure S3). *B. longum* 420 significantly increased IL‐12–producing cells, Granzyme B–producing CD8^+^ T cells, and IFN‐γ‐ and TNF‐α‐producing CD4^+^ and CD8^+^ T cells compared with PBS. CD69^+^ and IL‐2^+^ CD4^+^ T cells as well as CD69^+^ CD8^+^ T cells showed an increasing trend in the *B. longum* 420 group. Data are shown as mean ± SEM (n = 5 per group). Statistical significance was assessed by two‐sided Student's t‐test. ^*^ *p* < 0.05, ^**^ *p* < 0.01.

### 3.2 *B. longum* 420 Enhanced CTL‐mediated Cytotoxic Activity Against Pan02 Cells

Splenocytes from *B. longum* 420‐vaccinated mice demonstrated enhanced cytotoxicity against Pan02 cells. Significant increases in specific lysis were observed at effector/target ratios of 80:1 and 10:1 (*p <* 0.05, *p <* 0.01). At a cell ratio of 40:1, the *B. longum* 420 group also tended to show higher activity (p = 0.050) (Figure 2).

**FIGURE 2:**
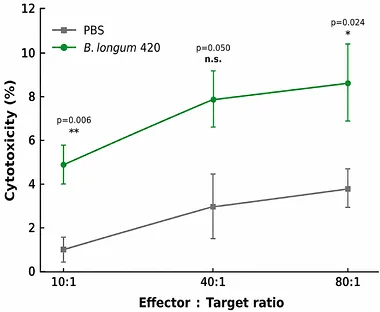
Cytotoxic Activity of Splenocytes After Oral Vaccination of *B. longum* 420. Splenocyte cytotoxicity against Pan02 cells was evaluated at the indicated effector‐to‐target (E:T) ratios. Splenocytes from *B. longum* 420‐treated mice exhibited significantly higher cytotoxic activity than those from PBS‐treated mice at E:T ratios of 10:1 and 80:1 (^*^ *p* < 0.05, ^**^ *p* < 0.01), with a trend toward increase at 40:1 (p = 0.050). Data are shown as mean ± SEM; each dot represents one mouse (n = 5 per group). Statistical significance was assessed by a two‐sided Student's t‐test at each E:T ratio.

### 3.3 *B. longum* 420 Combined with Anti–PD‐1 and Anti‐CTLA‐4 Antibody Showed Augmented Antitumor Effects and Improved Survival in the Pan02 Mouse PDAC Model

Combination therapy with *B. longum* 420 and ICIs exerted superior antitumor efficacy in vivo (Figure 3a). By day 31, tumor growth was significantly suppressed in the combination group compared with the PBS and ICI monotherapy groups (*p <* 0.05, *p <* 0.01). In addition, the *B. longum* 420 group also showed significantly suppressed tumor growth compared with the PBS group (*p* < 0.05) (Figure 3b). In a separate independent survival experiment (n = 7 mice per group), Kaplan–Meier analysis showed improved survival in the ICI‐treated groups, with the greatest benefit observed in the *B. longum* 420 + ICI combination group (Figure 3c). To assess antigen specificity, we included a WT1‐negative control strain (*B. longum* 2012) prepared and administered identically. The *B. longum* 2012 + ICIs group did not show additional tumor growth suppression compared with PBS + ICIs, whereas *B. longum* 420 + ICIs showed a stronger antitumor effect. In the same independent experiment, body weight was monitored throughout the treatment period, and no marked weight loss was observed in any group (Figure S5). In this independent experiment, ICIs were administered using anti‐mouse PD‐1 (clone RMP1‐14) and anti‐mouse CTLA‐4 antibodies (clone 9D9) (InvivoGen, San Diego, CA, USA) at a dose of 10 µg each.

**FIGURE 3:**
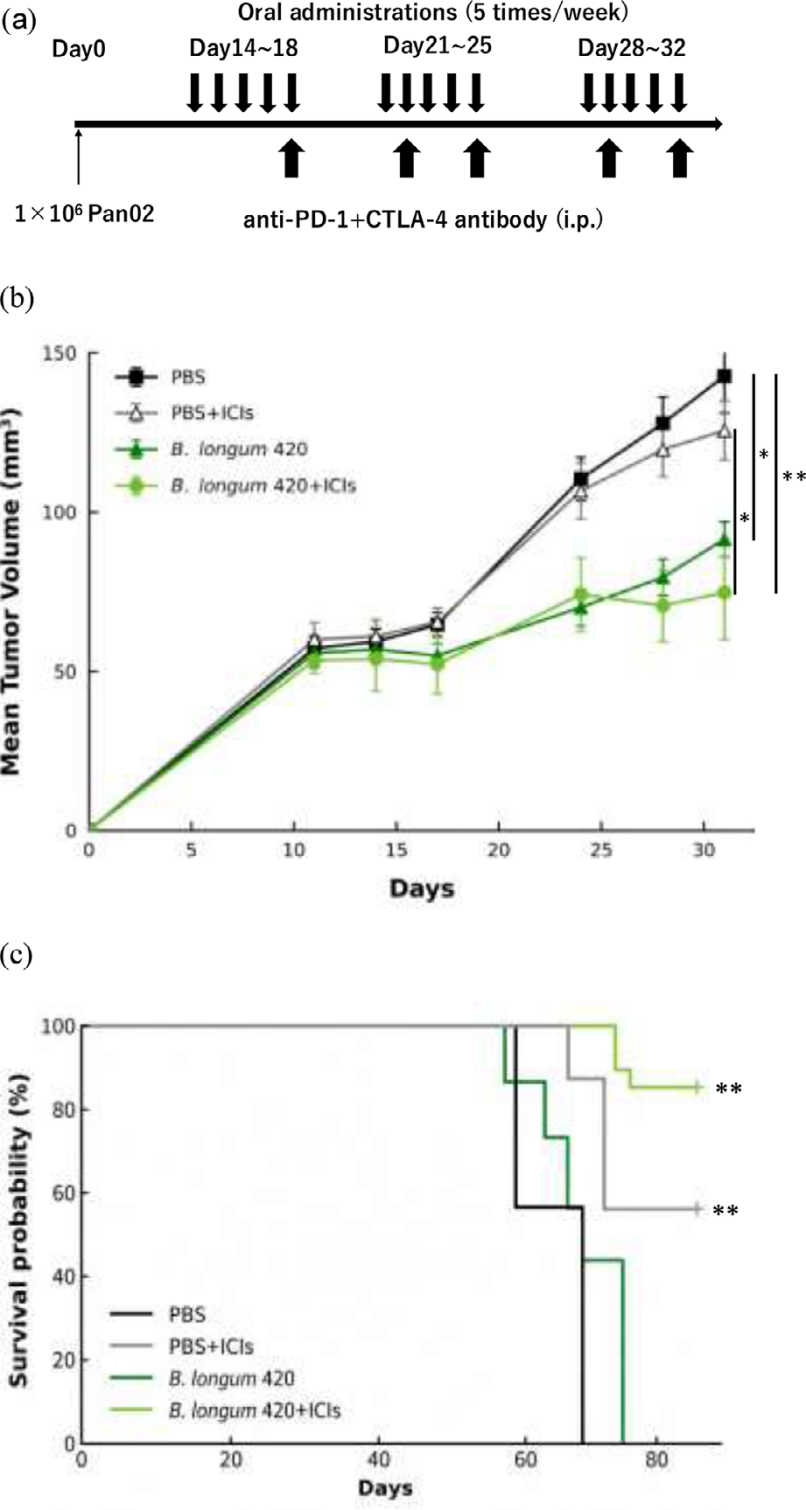
Antitumor effect of oral vaccination of *B. longum* 420 and ICIs against Pan02. (a) Schematic of the tumor challenge and treatment schedule. Pan02 cells were subcutaneously inoculated into mice. Starting on day 14, mice received PBS or *B. longum* 420 by oral administration five times per week for three weeks. ICI treatment (anti–PD‐1 + anti–CTLA‐4) was administered twice per week from day 15 for a total of six doses. (b) Tumor growth curves. Combination therapy (*B. longum* 420 + ICIs) significantly suppressed tumor growth compared with PBS and PBS + ICIs at day 31. *B. longum* 420 alone also suppressed tumor growth compared with PBS. Data are shown as mean tumor volume ± SEM (n = 10 or 11). Statistical significance at the indicated time point(s) was assessed by one‐way ANOVA with Tukey–Kramer multiple comparisons (two‐sided). ^*^ *p* < 0.05, ^**^ *p* < 0.01. (c) Kaplan–Meier survival curves (n = 7 mice per group). Statistical significance was assessed by a two‐sided log‐rank (Mantel–Cox) test. Combination therapy (*B. longum* 420 + ICIs) significantly improved survival compared with PBS (log‐rank test, p = 0.003).

### 3.4 Immunohistochemical Quantification of Intratumoral CD4^+^ and CD8^+^ T‐Cell Density in Pan02 Tumors

Immunohistochemical analysis with QuPath‐based quantification showed that the *B. longum* 420 + ICI group exhibited higher intratumoral CD8^+^ T‐cell density than the other groups. In contrast, CD4^+^ T‐cell density was variable among groups and was highest in the PBS group (Figure 4).

**FIGURE 4:**
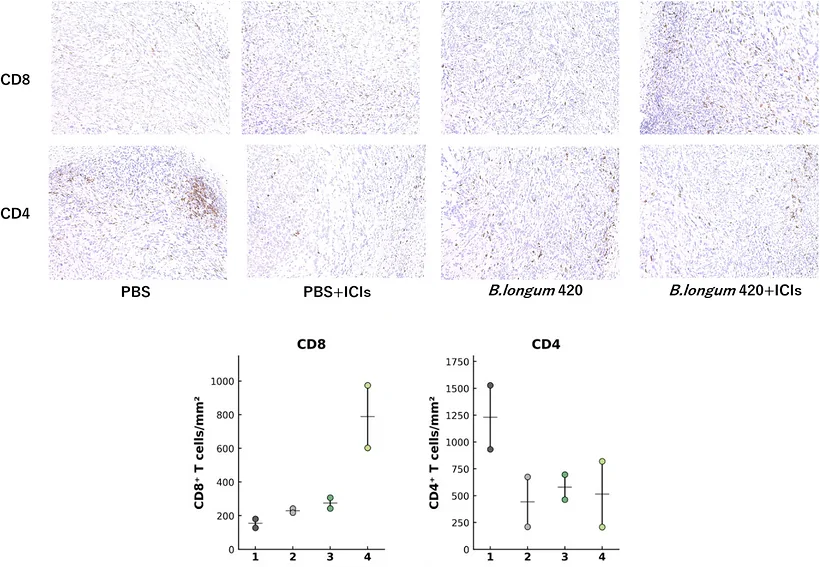
Immunohistochemical Staining for Tumor‐infiltrating T Cells in Pan02 Tumors. Resected Pan02 tumors were immunohistochemically stained with anti‐CD4 or anti‐CD8 antibodies. Representative images for each treatment group are shown (400×). CD4^+^ and CD8^+^ T cells were quantified as cells per square millimeter using QuPath. For each mouse, three randomly selected fields of view were imaged. In each image, the region of interest was defined as the entire tissue area within the image excluding blank background. Values were averaged across the three fields to obtain a single value per mouse. Individual mice are shown as dots. Bars indicate the group mean (n = 2 per group).

### 3.5 *B. longum 420* and ICIs Increased Activated CD8^+^ T‐Cell Infiltration and Altered CD4^+^ T‐Cell Subset Composition in Tumors

To further analyze tumor‐infiltrating CD8^+^ T cells, flow cytometry was performed. The combination therapy group with *B. longum* 420 and ICIs showed not only a simple increase in the proportion of CD8^+^ T cells compared with the other groups, but also significant increases in the proportions of CD44^+^CD62L^−^ effector T cells (Teff), CD44^+^CD62L^+^ central memory T cells (TCM), and PD‐1^+^ Ki‐67^+^ T cells, which is a cell proliferation marker (*p <* 0.05, *p <* 0.01). In contrast, the proportion of CD4^+^ T cells was highest in the PBS group. The proportions of CD4^+^CD25^+^FoxP3^+^ Treg cells and CD4^+^CD25^+^FoxP3^+^T‐bet^+^ Th1‐Treg cells were also higher in the PBS group than in the other groups. The proportion of CD4^+^FoxP3^−^T‐bet^+^ Th1 cells was highest in the PBS + ICI group (Figure 5) (Figures S6 and S7). To complement the proportion‐based analysis, we calculated absolute numbers of tumor‐infiltrating T‐cell subsets normalized to tumor weight. While the absolute counts showed similar trends, the differences were not statistically significant due to inter‐mouse variability (Figure S8).

**FIGURE 5:**
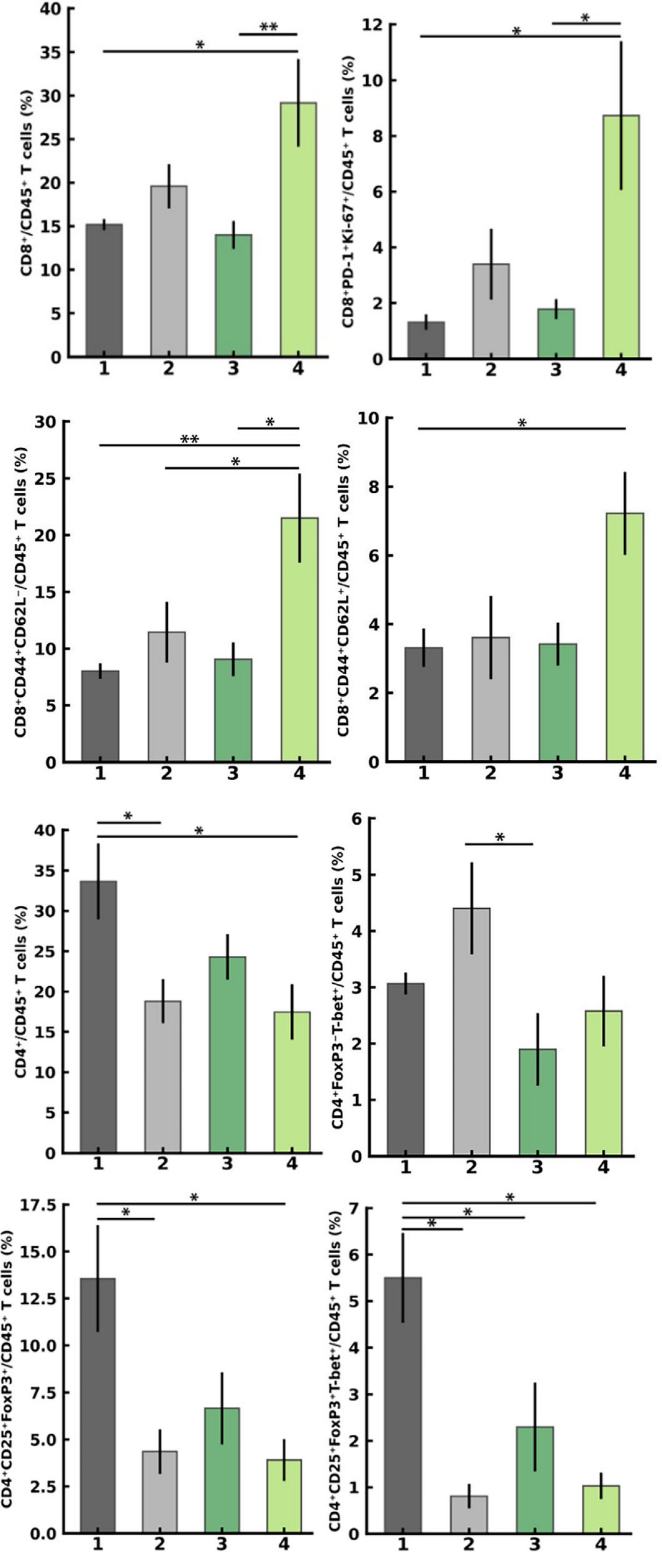
Evaluation of the Tumor Infiltrating Lymphocyte in Experiment Using Anti‐PD‐1 and Anti‐CTLA‐4 Antibody for Immune Checkpoint Inhibitor. Flow cytometric analysis of TILs showed that the combination of B. longum 420 with ICIs significantly increased the proportions of CD8^+^ T cells, CD8^+^ CD44^+^ CD62L^−^ effector T cells, CD8^+^ CD44^+^ CD62L^+^ central memory T cells, CD8^+^ PD‐1^+^ Ki‐67^+^ T cells, and CD4^+^ T cells, including CD4^+^ Foxp3^−^ T‐bet^+^ Th1 cells, CD4^+^ CD25^+^ Foxp3^+^ Treg cells, and CD4^+^ CD25^+^ Foxp3^+^ T‐bet^+^ Th1‐Treg cells, compared with other treatments. Data are shown as mean ± SEM (n = 7 or 6 per group). Statistical significance was assessed by one‐way ANOVA with Tukey–Kramer multiple comparisons (two‐sided). ^*^ *p* < 0.05, ^**^ *p* < 0.01. (gating strategy is shown in Figures S6 and S7).

## 4 Discussion

This study clarified the therapeutic impact of combining an oral cancer vaccine with ICI therapy for pancreatic cancer. Our results showed that the combination therapy of *B. longum* 420 with anti–PD‐1 and anti–CTLA‐4 antibodies significantly enhanced antitumor activity in a mouse PDAC model. Both the *B. longum* 420 monotherapy and combination therapy groups showed significant tumor growth suppression and improved survival compared with the PBS group (Figure 3). These findings indicate that although *B. longum* 420 alone possesses modest antitumor activity, its efficacy is maximized when combined with ICIs.

PDAC is known to be highly resistant to ICI monotherapy due to an immunosuppressive tumor microenvironment [2, 3, 4, 5, 6, 7, 8]. The observation that combination therapy overcame this barrier is therefore clinically meaningful. Many patients exhibit primary resistance or develop acquired resistance after an initial response. Development of new strategies to overcome these resistance mechanisms remains critically important for PDAC patients. Our findings support the possibility that combining a cancer vaccine with ICIs may address this challenge. Recent work has also explored engineered probiotics as oral antigen delivery platforms in PDAC. For example, an engineered probiotic strategy for oral delivery of a PDAC associated antigen has been reported [22].

One important finding of this study is that the oral cancer vaccine induced robust WT1 specific cellular immune responses. *B. longum* 420 significantly increased cytokine production—IFN‐γ, IL‐2, TNF‐α, and Granzyme B in splenocytes from immunized mice compared with the control group (Figure 1). IFN‐γ and IL‐2 are representative Th1 cytokines, and TNF‐α and Granzyme B, particularly secreted by CTLs, are known to play critical roles in the direct antitumor activity of cellular immunity [23, 24, 25]. These results, in addition to the findings from our previous study [21], confirm the increase in Granzyme B, an intracellular granule component of CTLs, further supporting that *B. longum* 420 activates WT1‐specific cellular immunity in vivo.

In addition, splenic ICCS showed an increased frequency of IL‐12 positive cells within the CD45 positive compartment in the vaccinated group. IL‐12 is a central mediator of type 1 immunity that links innate activation to Th1 polarization and the establishment of cytotoxic T cell responses [26]. Moreover, productive antitumor immunity under checkpoint blockade depends on dendritic cell and T cell crosstalk involving IFN‐γ and IL‐12, indicating that activation of this axis is associated with therapeutic responsiveness [27]. Therefore, the IL‐12 associated shift observed in the vaccinated group is consistent with the formation of a type 1 conditioned immune milieu that may support the induction and amplification of cellular immunity.

As a plausible upstream route for such immune induction, oral vaccines are expected to initiate immune priming in gut associated lymphoid tissues. Peyer's patches and M cell containing follicle associated epithelium are recognized as inductive sites that facilitate luminal antigen sampling and delivery to antigen presenting cells [28, 29]. In the present study, we did not directly evaluate Peyer's patches or mesenteric lymph nodes, and we cannot conclude dendritic cell activation in these sites from our data alone. Future studies assessing antigen uptake and immune activation in these tissues will be needed to directly test the intestinal priming mechanism.

Another key finding is that the combination therapy may have shifted the immunologically cold PDAC tumor microenvironment toward a more immunologically active state. Immunohistochemical quantification showed that intratumoral CD8^+^ T cell density was higher in the combination therapy group than in the other groups, whereas CD4^+^ T cell density was variable across groups and was highest in the PBS group (Figure 4). These results indicate that interpretation of the CD4 compartment requires attention not only to overall abundance but also to subset composition.

Flow cytometric profiling demonstrated that treatment associated differences in the intratumoral CD4 compartment were better captured by CD4 subset composition. The PBS group showed higher proportions of regulatory CD4 subsets including CD4^+^ CD25^+^ FoxP3^+^ Treg cells and CD4^+^ CD25^+^ FoxP3^+^ T‐bet^+^ Th1‐Treg cells (Figure 5). First, an increased proportion of Treg and Th1‐Treg subsets can contribute to a lack of antitumor efficacy by suppressing effector function within the tumor microenvironment and by constraining the induction and expansion of effective CD8 responses. T‐bet expressing regulatory T cells can be maintained and remain functional during type 1 inflammation [30]. In addition, CXCR3 expressing regulatory T cells have been shown to interact with type I dendritic cells in tumors and restrict CD8^+^ T cell antitumor immunity [31]. Conceptual discussion of Th1 like Treg populations further supports the idea that such regulatory states can limit antitumor immunity even in type 1 polarized settings [32]. Taken together, the relative enrichment of Treg and Th1‐Treg subsets in the PBS group is consistent with an immunosuppressive baseline state that is unfavorable for effective tumor control.

Second, the PBS + ICI group showed the highest proportion of CD4^+^ FoxP3^−^ T‐bet^+^ Th1 cells (Figure 5). However, Th1 differentiation within the CD4 compartment does not necessarily translate into antitumor efficacy when robust CD8 induction or expansion is not established. CD4 help is important for the development of CD8 responses in cancer immunology and immunotherapy [33], and this concept is supported across contexts of chronic infection and cancer [34]. Mechanistically, CD4 derived help is often relayed through dendritic cell licensing programs that enhance cross presentation and provide costimulation and cytokine support to CD8^+^ T cells [35]. In addition, productive antitumor immunity under checkpoint blockade requires dendritic cell and T cell crosstalk involving IL‐12 [27]. Therefore, even when Th1 skewing is observed, insufficient activation of dendritic cell licensing and IL‐12 linked pathways may limit intratumoral CD8 induction and expansion and thereby prevent the emergence of measurable antitumor efficacy [27, 33, 35]. This framework supports the interpretation that Th1 expansion alone is a necessary but not sufficient condition for effective CD8 mediated tumor control.

In contrast, the combination therapy induced not only an increase in the proportion of CD8^+^ T cells but also qualitative changes in these cells. The combination group showed significant increases in Teff, TCM, and PD‐1^+^ Ki‐67^+^ CD8^+^ T cells, which indicate proliferative and activation associated features (Figure 5). Teff cells are considered a correlate of survival benefit in immunotherapy responsive cancers, yet they are rarely observed in the PDAC tumor microenvironment [36]. Taken together, these findings suggest that the oral WT1 cancer vaccine combined with ICIs strengthens intratumoral CD8 activation, proliferation, and memory associated features, which is consistent with the enhanced antitumor efficacy observed in this model.

In this study, the proportion of PD‐1^+^ Ki‐67^+^ CD8^+^ T cells was significantly increased. In multiple tumor types, most tumor infiltrating CD8^+^ T cells express PD‐1, suggesting that PD‐1 is not merely a marker of exhaustion but a characteristic of tumor specific T cells that have been exposed to antigen and recognize tumors [37, 38]. Regarding Ki‐67, it has been reported that the proportion of PD‐1^+^ Ki‐67^+^ CD8^+^ cells in peripheral blood increases after anti‐PD‐1 treatment, and that patients with an early response show clinical benefit, whereas those with delayed or absent responses have limited therapeutic effects [39]. In the present study, the increase in PD‐1^+^ Ki‐67^+^ cells in tumor‐infiltrating T cells one week after treatment suggests that PD‐1 and Ki‐67 expression on intratumoral CD8^+^ T cells may be associated with antitumor efficacy. Prior phenotypic analyses reported that PD‐1^+^ Ki‐67^+^ CD8^+^ T cells exhibit an effector‐like phenotype [39]. Consistently, CD44^+^CD62L^−^ effector‐type cells showed an increasing trend among the expanded population in our study (Figure S6), suggesting a direct association with enhanced antitumor effects. It has also been reported that these cells can highly express CTLA‐4 [39], which may contribute to the high efficacy of the triple combination therapy with *B. longum* 420, anti‐PD‐1 antibody, and anti‐CTLA‐4 antibody.

We also observed an increase in TCM, a subset known for long‐term persistence and high self‐renewal capacity that contributes to durable therapeutic responses in cancer immunotherapy [40, 41]. In addition, interaction with tissue resident memory T cells is important for long‐term antitumor effects [42]. It has been proposed that precursors of tissue resident memory T cells can arise from TCM, and that TCM can efficiently generate tissue resident memory T cells due to high plasticity and tissue adaptability [43]. The significant increase in TCM suggests that a portion of these cells may differentiate into tissue resident memory T cells. This mechanism could help maintain therapeutic effects over time, even after completion of combination therapy.

Several limitations of this study warrant mention. First, the Pan02 model does not fully recapitulate features of human PDAC and likely exhibits limited fibrosis. Because dense fibrosis in PDAC can act as a barrier to drug delivery and immune cell infiltration, validation in more fibrosis‐rich models is needed. Evaluation using patient‐derived xenograft models or genetically engineered mouse models such as the KPC model is expected to advance clinical translation. Second, tumor rechallenge experiments were not performed, and persistence of vaccine‐induced immunity and effects on recurrence remain unverified. Although increased TCM suggests formation of long‐term immunological memory, long‐term follow‐up studies and rechallenge experiments will be required. Third, gastrointestinal residence of the administered bacteria was not directly measured by methods such as fecal CFU quantification or strain‐specific qPCR, which limits interpretation of dosing durability and mechanism. Because the bacteria were heat inactivated, colonization is not expected under our conditions. Fourth, we did not assess immune responses in Peyer's patches or mesenteric lymph nodes, which are key sites for intestinal priming after oral administration. Future studies will evaluate antigen uptake and immune activation in these tissues to clarify the mechanism of action. Fifth, we monitored body weight during treatment and observed no marked body weight loss. This finding supports tolerability in the short term. However, histopathological examination of major organs was not performed, and subclinical toxicities cannot be excluded without further evaluation.

## 5 Conclusions

This study confirmed that *B. longum* 420 can induce WT1‐specific T cell responses in C57BL/6J mice, and further demonstrated that combination with anti‐PD‐1 and anti‐CTLA‐4 antibodies induces a greater number of tumor‐infiltrating lymphocytes. These results indicate that, similar to the urothelial carcinoma and prostate cancer revealed in our previous studies, combination therapy with *B. longum* 420 and ICIs may represent a promising strategy for PDAC. This study demonstrates that the combination of an oral WT1‐targeted cancer vaccine and immune checkpoint inhibitors represents a promising therapeutic strategy for PDAC, which has traditionally been resistant to immunotherapy, and provides a strong rationale for further preclinical optimization and clinical development.

## Author Contributions

Research conception and design: **Taiki Yamazaki**, **Aori Minami**, and **Toshiro Shirakawa**. Data acquisition: **Taiki Yamazaki**, **Aori Minami**, **Misako Hirota**, **Mirei Hirano**, **Ryota Sato**, and **Atsuki Honma**. Statistical analysis: **Taiki Yamazaki** and **Aori Minami**. Data analysis and interpretation: **Taiki Yamazaki** and **Aori Minami**. Drafting of the manuscript: **Taiki Yamazaki**. Writing of the manuscript: **Taiki Yamazaki** and **Toshiro Shirakawa**. Critical revision of the manuscript: **Toshiro Shirakawa** and **Hideto Ueki**. Obtaining funding: Toshiro Shirakawa. Administrative, technical, or material support: None. Supervision: **Toshiro Shirakawa**. Approval of the final manuscript: **All authors**.

## Funding

This study was supported by the Translational Research Program, Seeds C, of the Japan Agency for Medical Research and Development (AMED) under Grant Numbers JP22ym0126081h0001, JP23ym0126081h0002, and JP24ym0126081h0003.

## Ethics Statement

All experiments and methods were performed in accordance with the relevant guidelines and regulations, and all experimental protocols, including animal experimental designs and procedures, were reviewed and approved by the Institutional Ethics and Animal Welfare Committees of the Kobe University Graduate School of Medicine. This study is reported in accordance with the ARRIVE Guidelines (https://arriveguidelines.org).

## Conflicts of Interest

Toshiro Shirakawa serves as Chief Executive Officer of Immunorock Co., Ltd. The other authors declare no conflicts of interest related to this work.

## Supporting information




**Supporting File 1**: advs76395‐sup‐0001‐FigureS1‐S8.pptx.

**Supporting File 2**: advs76395‐sup‐0002‐DataFiles.zip.

## Data Availability

The data that support the findings of this study are available from the corresponding author upon reasonable request.
