# Supplementary material for: WT1‐Targeted Oral Bifidobacterium longum Vaccine Enhances Checkpoint Blockade Efficacy in Pancreatic Cancer
Source: Adv Sci (Weinh). 2026 Jul 7:e24323. Online ahead of print. doi: 10.1002/advs.202524323 (PMC13338970; doi:10.1002/advs.202524323)
Supplement: Supplementary file 1 — Supporting File 1: advs76395‐sup‐0001‐FigureS1‐S8.pptx. [file ADVS-9999-e24323-s002.pptx]

## Slide 1
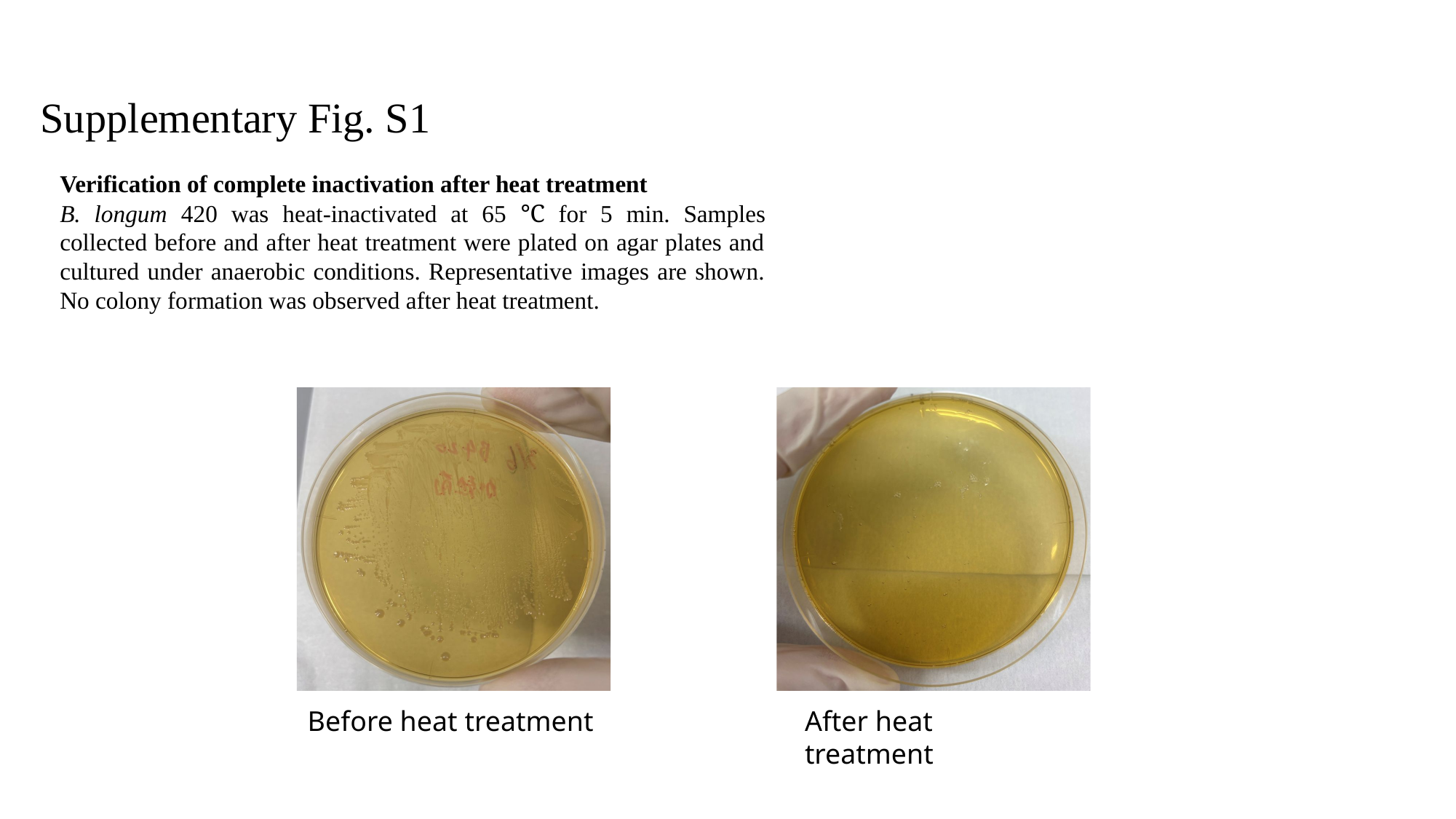

# Supplementary Fig. S1
Verification of complete inactivation after heat treatment
B. longum 420 was heat-inactivated at 65 ℃ for 5 min. Samples collected before and after heat treatment were plated on agar plates and cultured under anaerobic conditions. Representative images are shown. No colony formation was observed after heat treatment.
Before heat treatment
After heat treatment

## Slide 2
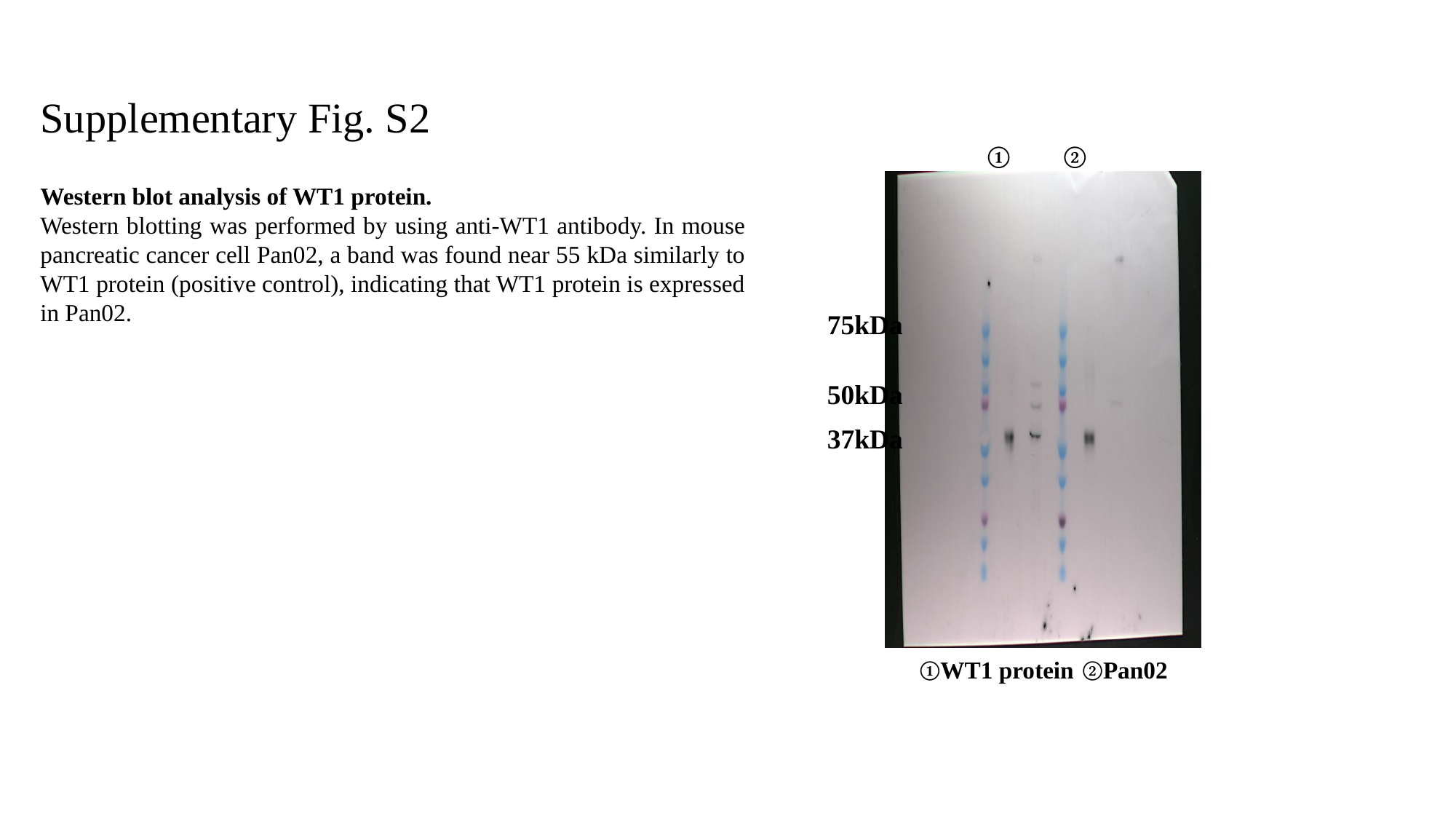

# Supplementary Fig. S2
①
②
Western blot analysis of WT1 protein.
Western blotting was performed by using anti-WT1 antibody. In mouse pancreatic cancer cell Pan02, a band was found near 55 kDa similarly to WT1 protein (positive control), indicating that WT1 protein is expressed in Pan02.
75kDa
50kDa
37kDa
①WT1 protein
②Pan02

## Slide 3
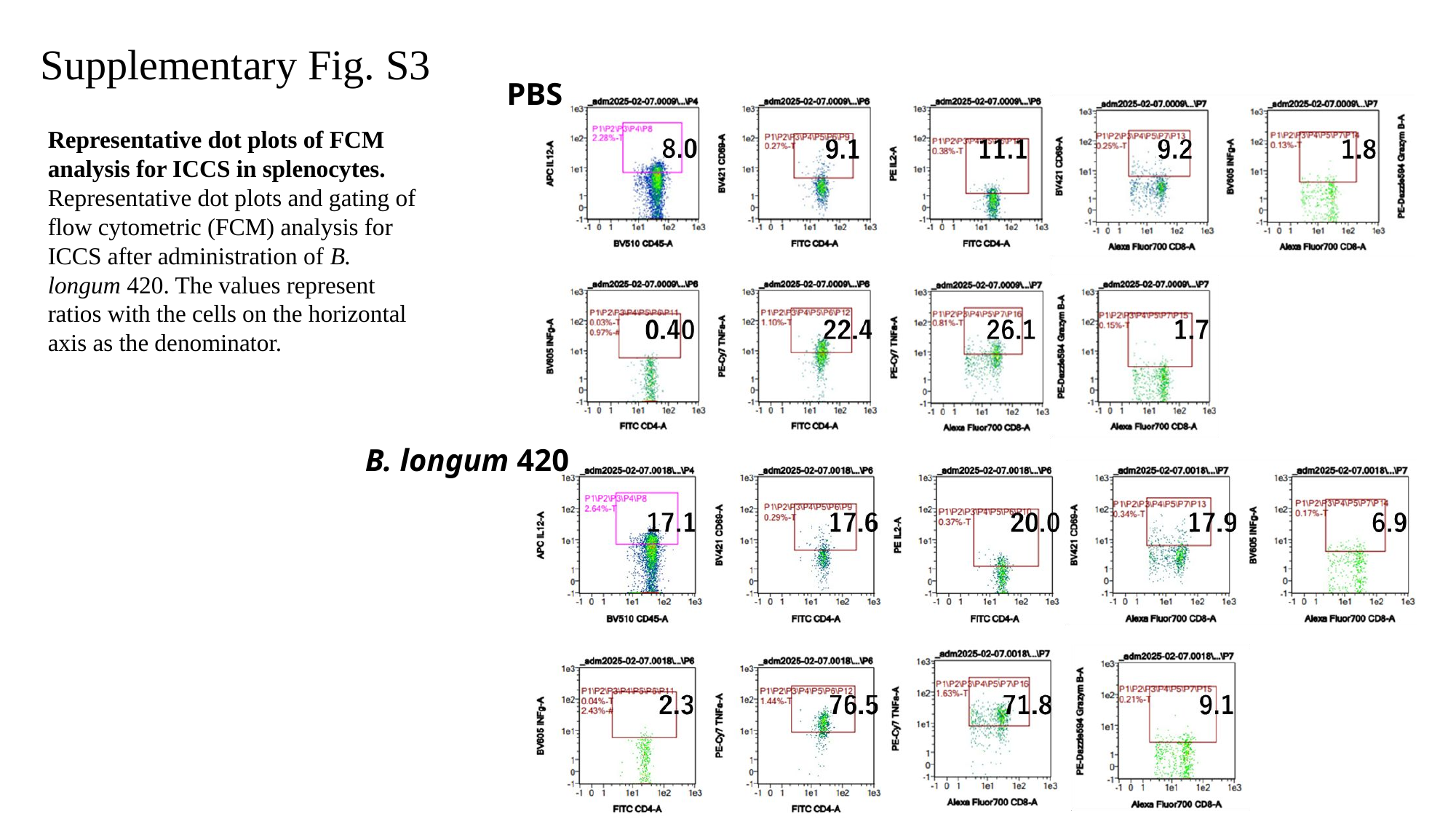

Supplementary Fig. S3
PBS
Representative dot plots of FCM analysis for ICCS in splenocytes.
Representative dot plots and gating of flow cytometric (FCM) analysis for ICCS after administration of B. longum 420. The values represent ratios with the cells on the horizontal axis as the denominator.
B. longum 420

## Slide 4
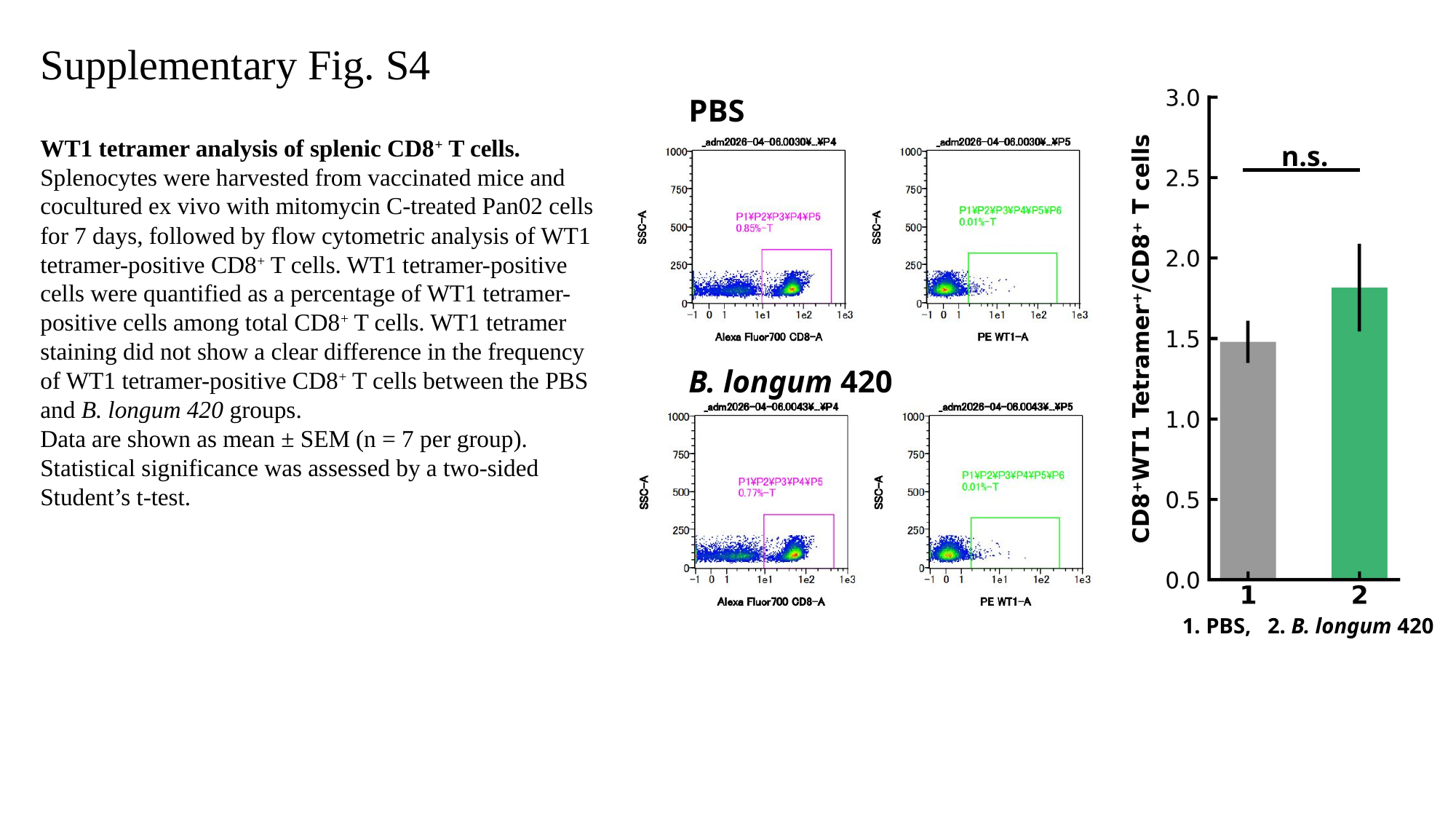

Supplementary Fig. S4
n.s.
PBS
WT1 tetramer analysis of splenic CD8+ T cells.
Splenocytes were harvested from vaccinated mice and cocultured ex vivo with mitomycin C-treated Pan02 cells for 7 days, followed by flow cytometric analysis of WT1 tetramer-positive CD8+ T cells. WT1 tetramer-positive cells were quantified as a percentage of WT1 tetramer-positive cells among total CD8+ T cells. WT1 tetramer staining did not show a clear difference in the frequency of WT1 tetramer-positive CD8+ T cells between the PBS and B. longum 420 groups.
Data are shown as mean ± SEM (n = 7 per group). Statistical significance was assessed by a two-sided Student’s t-test.
B. longum 420
1. PBS, 2. B. longum 420

## Slide 5
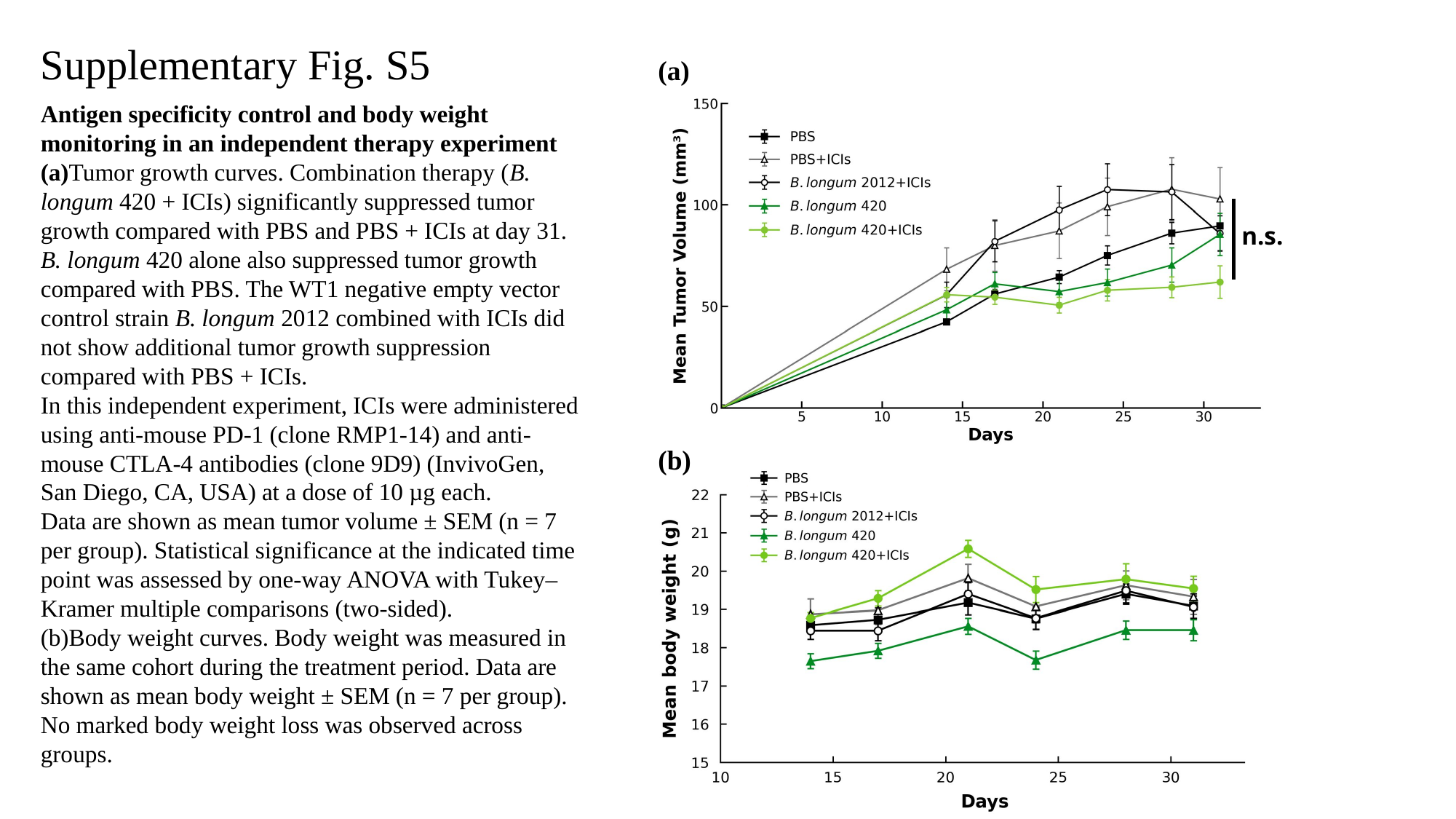

Supplementary Fig. S5
(a)
n.s.
Antigen specificity control and body weight monitoring in an independent therapy experiment
(a)Tumor growth curves. Combination therapy (B. longum 420 + ICIs) significantly suppressed tumor growth compared with PBS and PBS + ICIs at day 31. B. longum 420 alone also suppressed tumor growth compared with PBS. The WT1 negative empty vector control strain B. longum 2012 combined with ICIs did not show additional tumor growth suppression compared with PBS + ICIs.
In this independent experiment, ICIs were administered using anti-mouse PD-1 (clone RMP1-14) and anti-mouse CTLA-4 antibodies (clone 9D9) (InvivoGen, San Diego, CA, USA) at a dose of 10 µg each.
Data are shown as mean tumor volume ± SEM (n = 7 per group). Statistical significance at the indicated time point was assessed by one-way ANOVA with Tukey–Kramer multiple comparisons (two-sided).
(b)Body weight curves. Body weight was measured in the same cohort during the treatment period. Data are shown as mean body weight ± SEM (n = 7 per group). No marked body weight loss was observed across groups.
(b)

## Slide 6
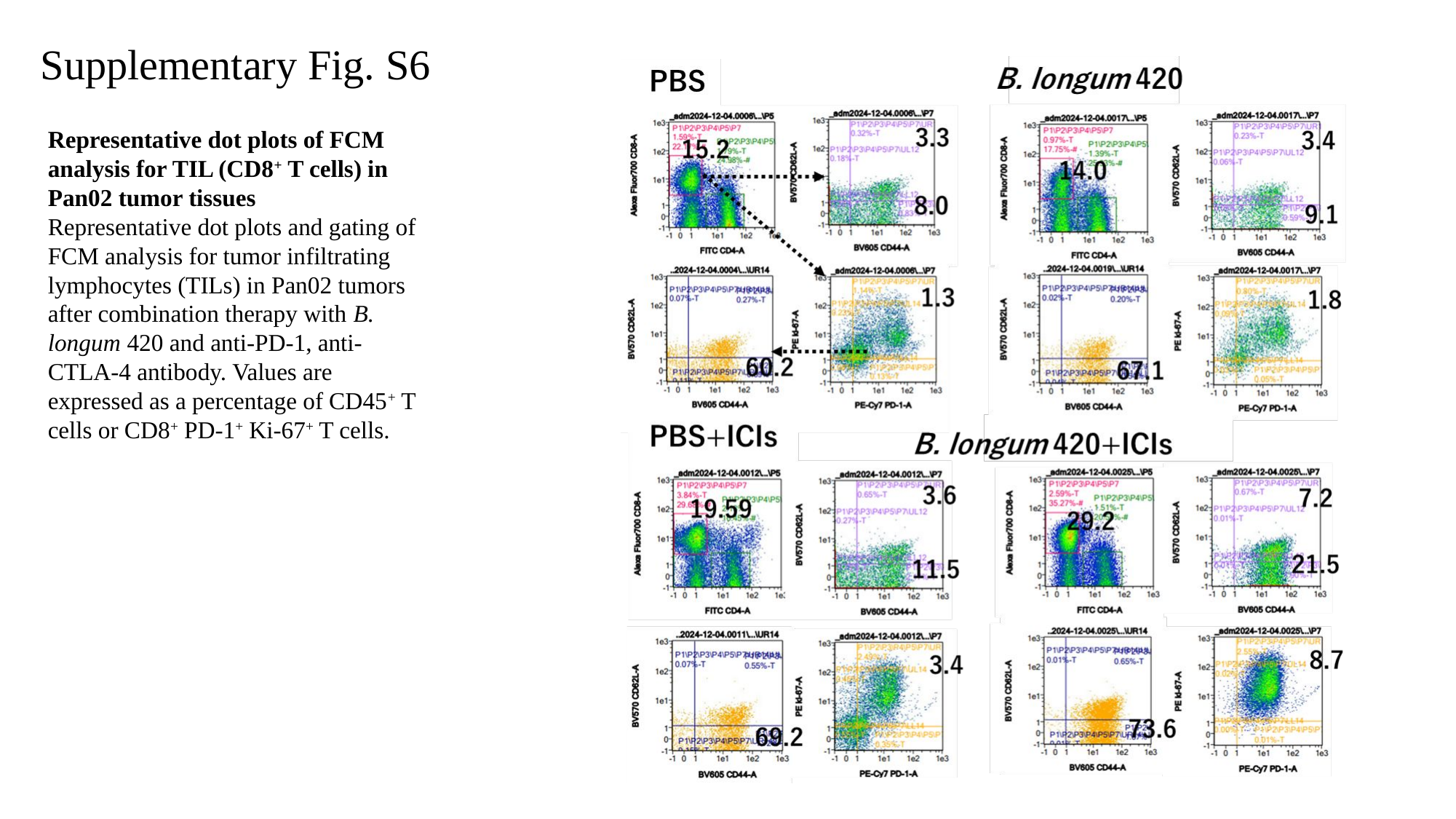

Supplementary Fig. S6
Representative dot plots of FCM analysis for TIL (CD8+ T cells) in Pan02 tumor tissues
Representative dot plots and gating of FCM analysis for tumor infiltrating lymphocytes (TILs) in Pan02 tumors after combination therapy with B. longum 420 and anti-PD-1, anti-CTLA-4 antibody. Values are expressed as a percentage of CD45+ T cells or CD8+ PD-1+ Ki-67+ T cells.

## Slide 7
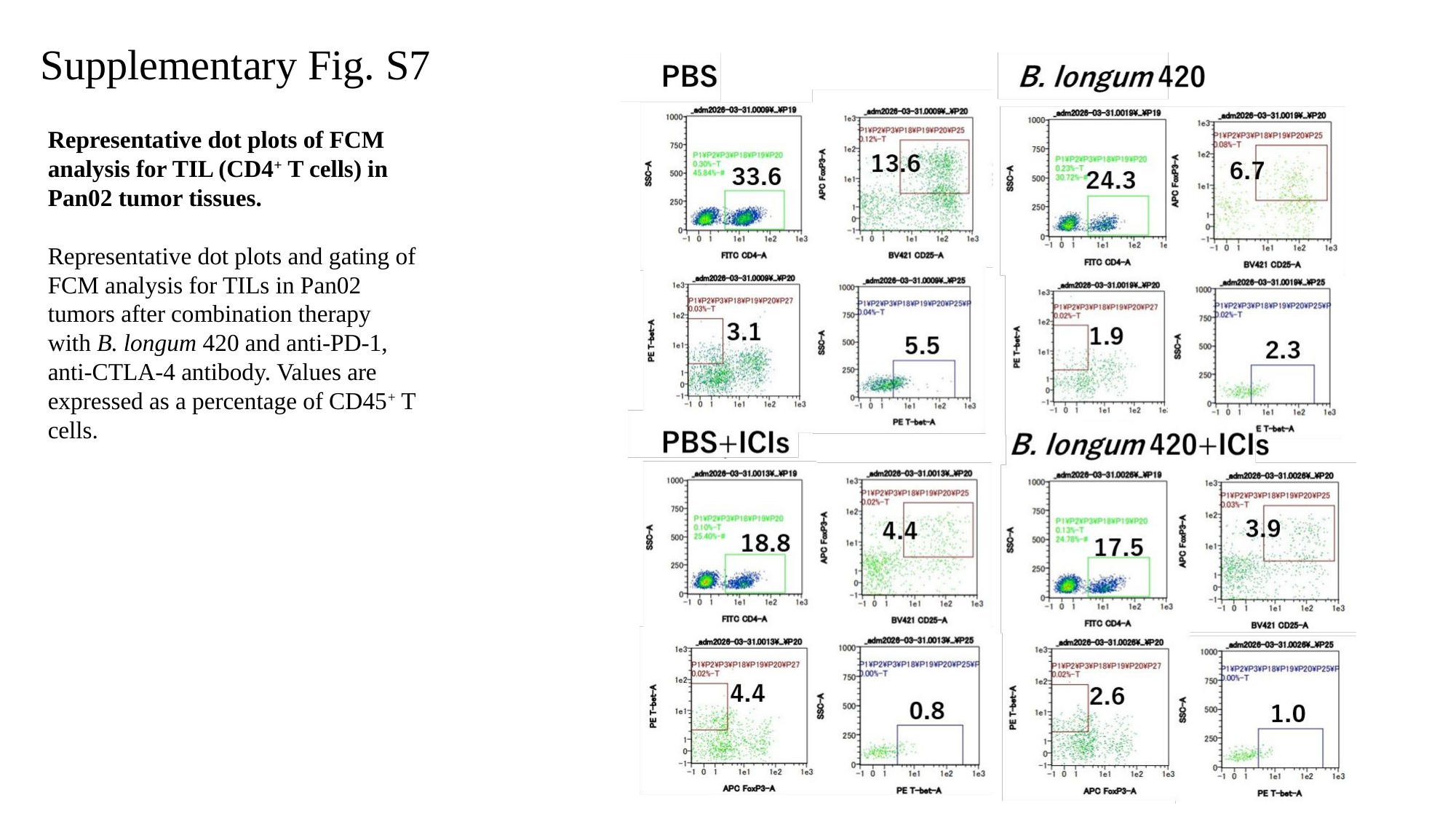

Supplementary Fig. S7
Representative dot plots of FCM analysis for TIL (CD4+ T cells) in Pan02 tumor tissues.
Representative dot plots and gating of FCM analysis for TILs in Pan02 tumors after combination therapy with B. longum 420 and anti-PD-1, anti-CTLA-4 antibody. Values are expressed as a percentage of CD45+ T cells.

## Slide 8
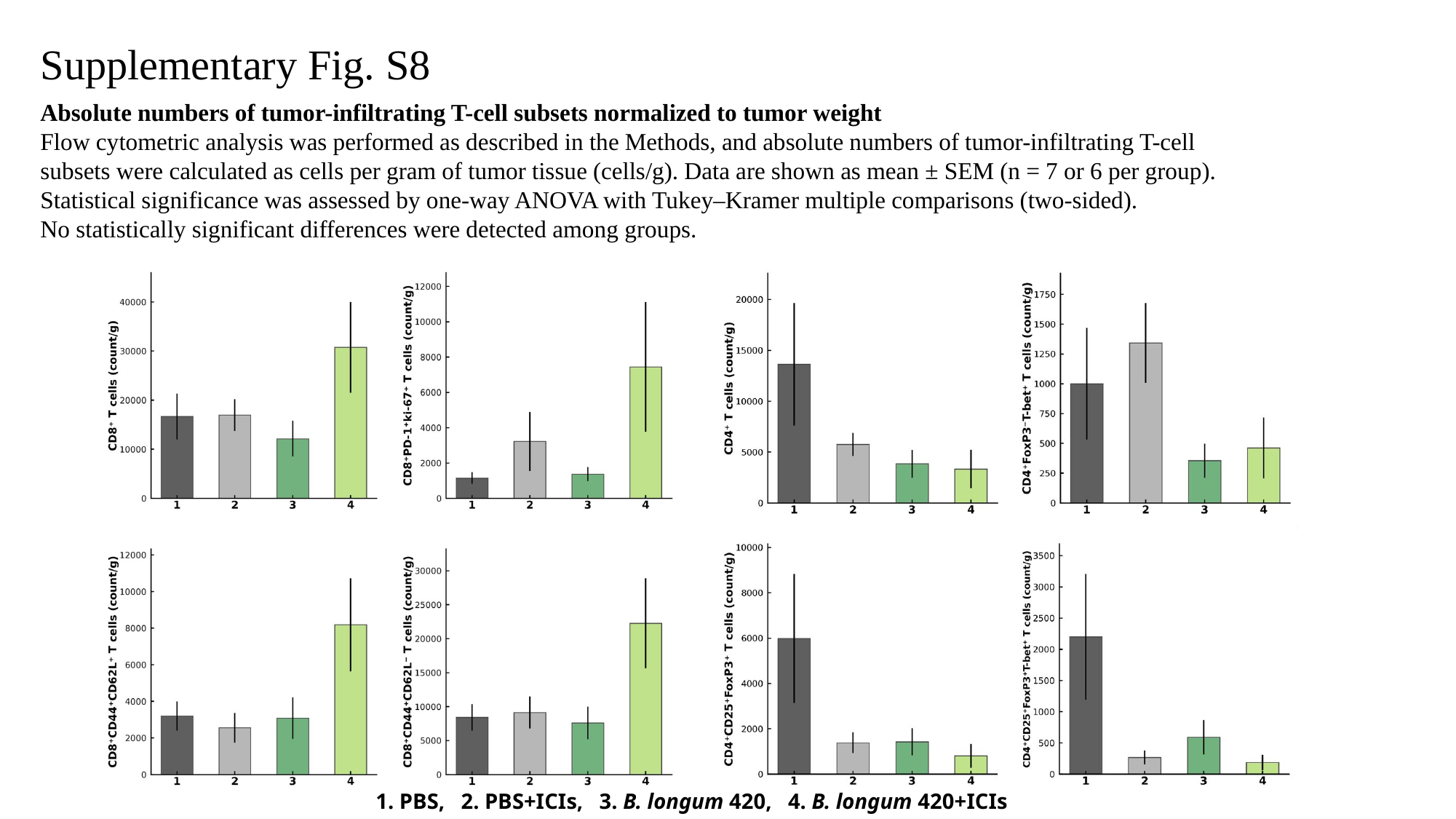

Supplementary Fig. S8
Absolute numbers of tumor-infiltrating T-cell subsets normalized to tumor weight
Flow cytometric analysis was performed as described in the Methods, and absolute numbers of tumor-infiltrating T-cell subsets were calculated as cells per gram of tumor tissue (cells/g). Data are shown as mean ± SEM (n = 7 or 6 per group). Statistical significance was assessed by one-way ANOVA with Tukey–Kramer multiple comparisons (two-sided).
No statistically significant differences were detected among groups.
1. PBS, 2. PBS+ICIs, 3. B. longum 420, 4. B. longum 420+ICIs
