# Supplementary material for: WT1‐Targeted Oral Bifidobacterium longum Vaccine Enhances Checkpoint Blockade Efficacy in Pancreatic Cancer
Source: Adv Sci (Weinh). 2026 Jul 7:e24323. Online ahead of print. doi: 10.1002/advs.202524323 (PMC13338970; doi:10.1002/advs.202524323)
Supplement: Supplementary file 2 — Supporting File 2: advs76395‐sup‐0002‐DataFiles.zip. [file ADVS-9999-e24323-s001.zip › Supplementary data legend.pdf]

Supplementary data legend

This document contains source data supporting the findings of the main manuscript.

Supplementary Data 1 : Source data for Figure 1.

This file contains the raw numerical data used to generate Figure 1 in the main manuscript, including the individual values for each marker shown in the figure.

Supplementary Data 2 : Source data for Figure 2.

This file contains the raw numerical data used to generate Figure 2 in the main manuscript, including the cytotoxicity values measured at each cell ratio.

Supplementary Data 3 : Source data for Figure 3b.

This file contains the raw data used to generate the mean tumor volumes shown in Figure 3b, including the measurements of the short and long diameters of individual tumors in each experimental group.

Supplementary Data 4 : Source data for Figure 3c.

This file provides the individual survival data used to generate the Kaplan–Meier survival curves shown in Figure 3c.

Supplementary Data 5 : Source data for Figure 5.

This file contains the raw data used to generate Figure 5, including the individual values for each marker shown in the figure.
